# Negative effects of psychotherapy: estimating the prevalence in a random national sample

**DOI:** 10.1192/bjo.2021.1025

**Published:** 2021-10-04

**Authors:** Bernhard Strauss, Romina Gawlytta, Andrea Schleu, Dominique Frenzl

**Affiliations:** Institute of Psychosocial Medicine, Psychotherapy, and Psychooncology, University Hospital Jena, Germany; Institute of Psychosocial Medicine, Psychotherapy, and Psychooncology, University Hospital Jena, Germany; Private Practice and Ethik Verein, Essen, Germany; Institute of Psychosocial Medicine, Psychotherapy, and Psychooncology, University Hospital Jena, Germany

**Keywords:** Negative effects, deterioration, helping alliance, psychotherapy, boundary violation

## Abstract

**Background:**

Negative or adverse effects of psychological treatments are increasingly a focus of psychotherapy research. Yet, we still know little about the prevalence of these effects.

**Aims:**

Starting from a representative national sample, the prevalence of negative effects and malpractice was determined in a subsample of individuals reporting psychotherapy currently or during the past 6 years.

**Method:**

Out of an initial representative sample of 5562 individuals, 244 were determined to have had psychotherapy within the past 6 years. Besides answering questions related to treatment, its effects and the therapists, patients filled out the Negative Effects Questionnaire, items of the Inventory of Negative Effects of Psychotherapy reflecting malpractice and the Helping Alliance Questionnaire, and rated psychotherapeutic changes in different areas.

**Results:**

Rates of positive changes related to therapy varied between 26.6% (relationship to parents) and 67.7% (improvement in depressed mood). Deteriorations were most commonly related to physical well-being (13.1%), ability to work (13.1%) and vitality (11.1%). Although patients generally reported a positive helping alliance, many of them reported high rates of negative effects (though not always linked to treatment). This was especially true of the experience of unpleasant memories (57.8%), unpleasant feelings (30.3%) and a lack of understanding of the treatment/therapist (19.3/18.4%). Indicators of malpractice were less common, with the exception that 16.8% felt violated by statements of their therapist.

**Conclusions:**

This study helps to better estimate aspects of negative effects in psychotherapy ranging from deteriorations, specific effects and issues of malpractice that should be replicated and specified in future studies.

Psychotherapy has proven to be a highly effective method to treat mental disorders and to improve patients’ social and general functioning, despite the percentage of remissions and clinically significant changes remaining limited. For several decades, psychotherapy and psychotherapy research were forced to justify their effects and to demonstrate cost-effectiveness, as both researchers and the public indicated some doubt about the efficacy of different psychotherapeutic treatments. The discussion and study of negative effects, side-effects, adverse effects or even harm have recently been intensified^1^ on a conceptual level, with respect to definitions^2,3^ and ways to grasp these effects in routine clinical practice.^4^ In addition, there has been an increase in research related to the occurrence of negative effects as well as their determinants in different treatment settings.^5,6^

Parry et al^1^ have spoken of the need for a new framework to proceed in this research field. One part of this framework must be a clearer differentiation of unwanted effects of psychotherapy,^7^ e.g. discriminating adverse or negative effects as a result of a regularly performed treatment from harmful and adverse effects due to unethical or unprofessional therapist behaviour. In addition, adverse effects of specific interventions should be discriminated from (sustained) deterioration or lasting negative effects.^1^ In any case, routine recording and reporting of negative effects in clinical practice, and in trials, needs to become more common.^8^ Scott and Young^9^ have stated that empirical research on negative effects is insufficient owing to a lack of a coherent framework for defining, discussing and monitoring such effects.

It is very difficult to get reliable information about the prevalence and occurrence of negative effects and harm as a consequence of psychological treatment.^9^ Specific measures of negative indicators and effects are just starting to become more common and widespread through clinical research.^4,10,11^ Sources of this information include reviews and meta-analyses of the general effects of the treatment of specific disorders, which commonly mention negative effects, but only marginally.^12^ Other studies discuss or review negative effects or harm more specifically^13–15^ and discuss the consequences of such effects for clinical practice and training.^16,17^

Studies specifically designed to estimate the prevalence of negative effects are still extremely rare. In a nationwide survey in England and Wales, Crawford et al^5^ asked individuals who had received psychotherapy whether they had experienced lasting negative effects. The result was that 763 (5.2%) out of a total sample of 14 587 respondents reported lasting negative effects. Crawford et al chose the strategy of approaching a large sample of psychotherapy patients. In their study, patients were treated in 184 different services.

Another way to estimate the prevalence of negative effects would be to survey a sample that is representative of the total national population and then select those individuals who have psychotherapy experience. Albani et al^18–20^ chose such a strategy to get information about general evaluations of patients related to their psychotherapeutic treatment. In one study,^19,20^ 46 686 individuals were screened for psychotherapeutic experiences within the past 6 years, with 1212 being identified and surveyed in detail. In a second study,^18^ 5120 individuals were screened and 7% (379) fulfilled the criterion. Unfortunately, this survey, although starting with a representative German sample, did not explicitly study any negative effects. The authors instead asked about changes in different symptoms and life areas, including the option of reporting no change or changes for the worse.

In the present study, following the template of the Albani survey, we screened a representative sample of a similar size to that used in Albani's second study (for financial reasons) to reach a final sample of approximately 250 individuals with psychotherapeutic experience. We aimed to replicate some treatment-related changes on a symptom level as well as more general effects on one's life. In addition, we asked for ratings of the therapeutic alliance and included a standardised questionnaire focusing on negative effects, as well as some items covering malpractice and boundary violations.

This study will also serve as a pilot survey, with a future goal to replicate it in a larger sample, probably with more specific questions related to the entire field of unwanted and unexpected effects of psychotherapy.

## Method

### Setting and participants

The study was planned in cooperation with a company specialising in market and social research (USUMA, Berlin), abiding by the German law of data protection (§30a BDSG, German law of protection of data privacy). The company was financed by the research team to draw a sample based on screening interviews and to perform the complete survey via telephone in a defined time period (1 July 2019 to 25 October 2019).

As the incidence of psychotherapy use in the sample was expected to be low, the survey included two subsamples.

**Fig. 1:**
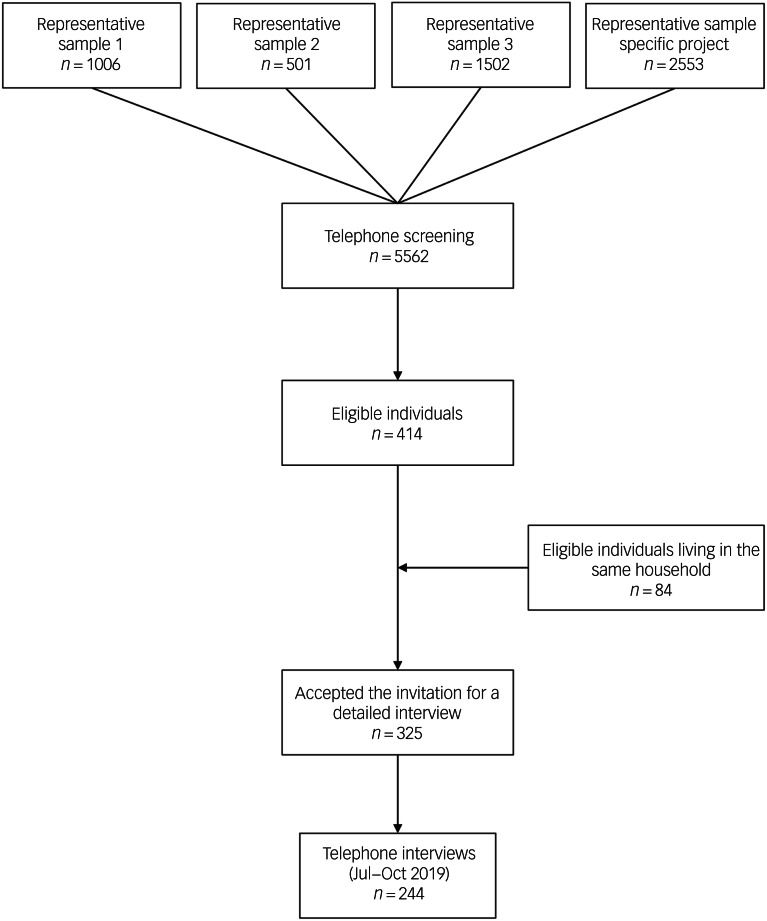
Flow of participants.

The total sample for the screening interviews consisted of 5562 individuals living in Germany with an age greater or equal to 18 years (Fig. 1). The screening questions were initially sent to *N* = 3009 individuals (54.1% of the total) via three independent nationwide representative social surveys. In addition, a specific recruiting project was performed combining the screening questions directly with the survey. Here, *N* = 2553 individuals were contacted (45.9% of the total).

The basic screening question was: ‘Have you had (or received) psychotherapeutic treatment during the last 6 years or are you currently in psychotherapy?’ Before asking this question, all participants received explanations of the term psychotherapy/psychotherapeutic treatment (i.e. the psychological treatment of mental problems) to avoid inaccuracy. A total of 414 (7.44%) answered positively. Another 84 mentioned that another adult person living in the same household would have psychotherapeutic experience during the same time range.

Invitations to be interviewed were sent and 325 individuals accepted. Finally, interviews (via telephone with an average duration of 20 min) were performed with a total of 244 individuals. Verbal consent of the participants was witnessed and formally recorded. The reduction to 244 participants was because of the limitation of the interview time period and the fact that some individuals withdrew their consent or were not available for the interview.

In general, all households were selected based on the ADM-telephone sample ‘Easy Sample’ (including all telephone numbers according to the Gabler/Haeder procedure) to ensure a random selection of the sample. Within a household, the indexed person was randomly selected as well (according to the Kish selection grid^21^). In summary, the sampling procedure first targeted random sample point areas, then a random household within those areas, and finally chose a person within these households.

### Measures

Participants reported their sex, age, educational level, marital status and monthly net household income. Information regarding the psychotherapeutic treatment (treatment setting [in-patient, out-patient, day treatment]; treatment modality [individual, group, couple or family therapy]; therapist profession [psychologist, medical doctor, other]; therapist gender; estimated age of the therapist; previous psychotherapy; additional pharmacotherapy) was also assessed.

Besides the variables used to describe the sample and the treatment, the survey provided a list of 24 problems that commonly lead to seeking a psychotherapist. These were similarly used in the survey of Albani et al.^19^ Each person was asked to rate (on a five-point scale) whether the problems improved (1 = much, 2 = somewhat), remained unchanged (3) or deteriorated (4 = somewhat, 5 = much).

We further used the 11 items of the Helping Alliance Questionnaire (HAQ),^22^ a common instrument used to rate aspects of the working alliance with the therapist. The HAQ measures the strength of the patient–therapist therapeutic alliance. Authors of the German version of the HAQ-11 reported an internal consistency of alpha (Cronbach's α) between 0.91 and 0.95^23^ for the two subscales related to satisfaction with therapeutic outcome and the quality of the relationship. In the present investigation, Cronbach's α values were 0.79 and 0.93 respectively.

The core instrument of the survey was the German short version of the Negative Effects Questionnaire (NEQ),^2^ the negative-effects-related questionnaire available in the most different languages (more than 10^4^). The NEQ covers 20 items describing negative treatment responses dealing with symptoms, quality, dependency, stigma and hopelessness. The answering schema first differentiates the occurrence of an effect (yes/no) and then asks for the perceived burden (five points) and its potential relation to treatment or other reasons (yes/no). The original NEQ comprises six factors/scales with internal consistencies ranging between alpha = 0.72 and 0.92. In the current investigation, Cronbach's α for the NEQ was 0.83. Since the NEQ does not include questions related to misconduct or malpractice, we also used the six items (rated on a four-point scale) from the Inventory for the Assessment of Negative Effects of Psychotherapy (INEP).^24^ Finally, seven items were included from the Albani survey^19^ related to the end of the treatment and the perceived competence of the therapist.

### Statistical methods

The statistical analyses were performed with IBM SPSS Statistics, version 26. Primarily, descriptive data and frequencies were examined. In order to determine possible differences between experiences from a current or a past psychotherapy, Pearson's χ^2^-tests were performed. When expected cell counts were less than five, we used Fisher's exact test instead. In case of significant differences, we also calculated Cramer's V in order to get an impression of the effect size.

When contrasting negative effect rates (NEQ) for patients with terminated treatment and those still in psychotherapy, we only included cases where negative effects were related to the treatment. Differences regarding the therapeutic alliance (HAQ) between treatment completers and patients still in psychotherapy were calculated using the Mann–Whitney U-test. To test the relationship between negative effects (number of negative effects related to therapy) and the subscales of the HAQ, we further calculated correlations.

### Ethics statement

All procedures contributing to this work complied with the ethical standards of the relevant national and institutional committees on human experimentation and with the Helsinki Declaration of 1975, as revised in 2008. All procedures involving human subjects/patients were approved by Ethics Committee of the University Hospital Jena (2020-1853-Bef).

## Results

### Sample characteristics

A comparison of a subsample of the eligible individuals who had already provided sociodemographic data during the screening interview (*n* = 374) and the participants in the final survey, with criteria of representativeness, revealed that psychotherapy patients under 45 years of age were only slightly underrepresented in the final sample, whereas the educational level was slightly higher in the recruited sample than in the final sample (Table 1).

**Table 1:** Characteristics of the sample of this study compared with the sample of Albani et al^19^ and the national German population

Sample	This study			Albani et al	National population
characteristic	Female	Male	Total		
*n* (%)	145 (59.4%)	99 (40.6%)	244	1212	83 167^a^
Female				873 (72.0%)	41 038 (49.3%)^a^
Male				339 (28.0%)	42 129 (50.7%)^a^
Age, *M* (s.d.)	55.9 (15.1)	53.8 (15.2)	55.1 (15.2)	47.2 (13.3)	−
Age distribution, *n* (%)					
<45 years	35 (24.1%)	22 (22.2%)	57 (23.4%)	482 (40.0%)	25 276 (30.4%)^a^
45–65 years	68 (46.9%)	59 (59.6%)	127 (52.0%)	617 (50.9%)	25 390 (30.5%^)^^a^
>65 years	42 (29.0%)	18 (18.2%)	60 (24.6%)	113 (9.3%)	17 070 (20.5%)^a^
Years of education					
9	27 (18.6%)	18 (18.1%)	45 (18.4%)	242 (20.0%)	19 884 (29.8%)^b^
10	54 (37.2%)	29 (29.3%)	83 (34.0%)	434 (35.8%)	20 469 (30.7%)^b^
12–13	20 (13.8%)	21 (21.1%)	41 (16.8%)	204 (16.9%)	23 206 (34.8%)^b^
>13 (university)	42 (29.0%)	30 (30.3%)	72 (29.5%)	325 (26.8%)	−
Currently working (yes)	67 (46.2%)	48 (48.5%)	115 (47.1%)	643 (53.1%)	40 852 (49.9%)^c^
Family status					
Married	48 (33.1%)	42 (42.4%)	90 (36.9%)	470 (38.8%)	35 473 (42.7%)^d^
Single	38 (26.2%)	34 (34.2%)	72 (29.5%)	328 (27.1%)	35 791 (43.0%)^d^
Separated	9 (6.2%)	4 (4.0%)	13 (5.3%)	58 (4.8%)	−
Divorced	26 (17.9%)	14 (14.1%)	40 (16.4%)	271 (22.4%)	6280 (7.6%)^d^
Widowed	22 (15.2%)	4 (4.0%)	26 (10.7%)	78 (6.4%)	5621 (6.8%)^d^
Monthly net household income					
<1500 €	35 (27.7%)	20 (22.5%)	55 (25.6%)	n.a.^e^	8414 (22.1%)^f^
1500–2500 €	42 (33.3%)	20 (22.5%)	62 (28.8%)		7757 (20.4%)^f^
>2500 €	49 (38.8%)	49 (55.1%)	98 (45.8%)		21 823 (57.4%)^f^

*M*, mean; n.a., not applicable.

a. German Microcensus 2019;^25^ general population; data in thousands; *n* = 83 166 711.

b. German Microcensus 2019;^26^ population (>20 years) in private households (excluding individuals living in shared/community accommodation); data in thousands; *n* = 66 666 000.

c. German Microcensus 2019;^27^ population in private households (excluding individuals living in shared/community accommodation); data in thousands; *n* = 81 848 000.

d. German Microcensus 2019;^28^ general population; data in thousands; *n* = 83 166 711.

e. Study used different categories not comparable with the others.

f. Federal Statistical Office of Germany;^29^ private households (excluding individuals living in shared/community accommodation); data in thousands; *n* = 37 993 000.

Sociodemographic characteristics of the final sample (*N* = 244) are summarised in Table 1 and contrasted with the sample of the Albani survey and available data related to the German population (2019).^25–29^ The sample was 59.4% female (*n* = 145). The mean age of the entire sample was 55.1 years (s.d. = 15.2). Individuals living in communities with fewer than 20 k inhabitants comprised 33.8% of the sample, whereas 19.3% lived in communities with 20–100 k inhabitants, and 44.3% lived in cities with more than 100 k inhabitants.

### Conditions of psychotherapy

Of the 244 individuals, 68.9% had had psychotherapy in the past 6 years, and 43% were currently in therapy. The vast majority reported having had out-patient treatment (88.5%); 11% had undergone in-patient or day treatment. Short-term treatment of less than 1 year in duration was reported by 63.4%. The vast majority mainly had individual sessions. Only 6% reported experiences with group psychotherapy. Additional psycho-pharmacotherapy was reported by 52.9% of the sample.

The survey asked about the primary psychological problems leading to contact with a psychotherapist: 41% (*n* = 100) reported anxiety, 77% reported mood disorders or symptoms, substance misuse was the primary reason in 8.6%, and symptoms of eating disorders were mentioned by 13.1%. The most common additional problems included psychosomatic complaints (50.8%), traumatic experiences during life (48%) and work-related problems (38.1%). A diagnosis of a personality disorder was mentioned by 9%.

Regarding the psychotherapies, 63.1% mentioned having met a female therapist, with a majority visiting a psychologist (69.2%). The estimated age of the therapist most commonly was 40–49 (41.8%) or 50–59 (31.1%) years. Reports of having already had one psychological treatment before the current or last one were made by 25.4%, while 29.9% even reported having more than one psychotherapy in the past.

### Evaluation of psychotherapy and therapeutic alliance

Table 2 summarises the rates of improvement/no change/deterioration related to different aspects of the (former) patients’ lives. Rates of *unchanged problems* (ratings 3 and 4) ranged between 23.0% (vitality) and 45.5% (sexual satisfaction). Problems with the *highest deterioration rates* were physical well-being (13.1%), ability to work (13.1%), vitality (11.1%), sexual problems (10.6%) and problems with self-esteem (10.3%). The highest *rates of positive change* (ratings 1 and 2) were found for depressed mood (67.7%), interpersonal problems (66.4%), vitality (65.6%), personal development (63.3%), coping with daily stress (63.1%) and understanding other people (62.3%). In an additional question, 47% of the individuals reported improvement of their physical health (4.1% deterioration, 47.5% no change).

**Table 2:** Influence of psychotherapy on different aspects of life (‘How did psychotherapy influence the following aspects?’); percentages of the sample of 244 (former) patients

	Better/much better (1/2)	Unchanged (3)	Worse/much worse (4/5)
Ability to engage in interpersonal relationships	57.4%	35.2%	5.3%
Work-related productivity	47.1%	28.3%	8.6%
Ability to work	44.3%	26.6%	13.1%
Dealing with daily hassles	63.1%	26.2%	9.9%
Physical well-being	56.6%	29.9%	13.1%
Joy of life	65.6%	23.0%	11.1%
Personal development	63.1%	27.0%	7.8%
Understanding of others	62.3%	32.0%	4.5%
Interacting with others	66.4%	28.7%	3.7%
Self-esteem and self-trust	59.0%	29.9%	10.3%
Improvement in depressed mood	67.6%	23.4%	7.0%
Satisfying sexual life	31.6%	45.5%	10.6%
Partner relationship	39.8%	29.9%	3.2%
Relationship to parents	26.6%	30.7%	6.5%
Interpersonal relations in a professional context	44.3%	27.9%	4.5%

In a selection of items from the study of Albani et al,^19,20^ the 139 individuals who already had finished treatment described their psychotherapy in retrospect. Major problems were *solved* in 55.5%, whereas 87.6% reported that they could *deal better* with their problems; 13.1% (*n* = 20) changed their psychotherapist during their treatment, and 19% dropped out owing to negative expectations of success. In 24.2% of cases, the end of the treatment was proposed by the therapist. The maximum number of sessions reimbursed by the health insurance was reached by 39.2%. At least 15% (*n* = 23) mentioned doubts related to the competence of their therapist.

The results related to the individuals’ experiences with their therapists and the therapeutic alliance (HAQ) are reflected in Supplementary Table 1 available at https://doi.org/10.1192/bjo.2021.1025. On average, 88.6% of individuals agreed with the HAQ items indicating a positive working alliance with the therapist. The item with lowest agreement was ‘I can/could see that I will solve the problems that lead me into treatment’, with a disagreement rate of 18.9%. We did not find differences between treatment completers and patients still in psychotherapy regarding the two subscales of the HAQ (therapeutic progress [*U* = 6450.00, *P* = 0.498] and the quality of the relationship [*U* = 6031.50, *P* = 0.169]).

### Negative effects

As can be seen from Table 3, the most common negative effect (in total and attributed to treatment) was the resurfacing of unpleasant memories (57.8% in the total sample). Of those reporting this negative effect, 68.8% related it to the psychological treatment. The experiences of sleep problems, stress and unpleasant feelings as well as feeling more worried were also commonly reported (between 27.9% and 36.9% in the total sample).

**Table 3:** Frequency of negative effects, mean level of negative effects and proportion of negative effects attributed to treatment

NEQ item	Effect occurred	Average degree (1–5) M (s.d.)	Related to treatment (if effect occurred)	Related to treatment (total sample)	Treatment already terminated (*n* = 139)	Currently in treatment (*n* = 105)
I had more problems with my sleep	36.9%	2.47 (1.08)	21.1%	7.8%	14 (10.1%)	5 (4.8%)
I felt like I was under more stress	30.7%	1.96 (1.18)	30.7%	9.4%	14 (10.1%)	9 (8.6%)
I experienced more anxiety	14.8%	1.88 (1.22)	33.3%	4.9%	8 (5.8%)	4 (3.8%)
I felt more worried	27.9%	1.71 (1.08)	32.4%	9.0%	**17 (12.2%)**	**5** (**4.8%)**
I experienced more hopelessness	16.4%	1.83 (1.34)	32.5%	5.3%	10 (7.2%)	3 (2.9%)
I experienced more unpleasant feelings	30.3%	2.00 (1.27)	55.4%	16.8%	29 (20.9%)	12 (11.4%)
I felt that the issue I was looking for help with got worse	8.6%	1.89 (1.45)	47.6%	4.1%	7 (5.0%)	3 (2.9%)
Unpleasant memories resurfaced	57.8%	2.10 (1.41)	68.8%	39.8%	**65** (**46.8%)**	**32** (**30.5%)**
I became afraid that other people would find out about my treatment	10.2%	1.79 (1.18)	24.0%	2.5%	3 (2.2%)	3 (2.9%)
I got thoughts that it would be better if I did not exist anymore and that I should take my own life	7.8%	205 (1.47)	15.8%	1.2%	2 (1.4%)	1 (1.0%)
I started feeling ashamed in front of other people because I was having treatment	6.6%	2.00 (1.00)	43.8%	2.9%	4 (2.9%)	3 (2.9%)
I stopped thinking that things could get better	13.5%	2.23 (1.38)	24.2%	3.3%	7 (5.0%)	1 (1.0%)
I started thinking that the issue I was seeking help for could not be made any better	16.0%	2.11 (0.98)	46.2%	7.4%	14 (10.1%)	4 (3.8%)
I think that I have developed a dependency on my treatment	7.0%	1.71 (0.99)	70.6%	4.9%	6 (4.3%)	6 (5.7%)
I did not always understand my treatment	19.3%	1.15 (1.05)	57.4%	11.1%	15 (10.8%)	12 (11.4%)
I did not always understand my therapist	18.4%	1.58 (1.05)	68.9%	12.7%	17 (12.2%)	14 (13.3%)
I did not have confidence in my treatment	11.5%	2.00 (1.30)	60.7%	7.0%	10 (7.2%)	7 (6.7%)
I felt that the treatment did not produce any results	11.1%	2.08 (1.44)	66.7%	7.4%	13 (9.4%)	5 (4.8%)
I felt that my expectations for the therapist were not fulfilled	20.9%	1.59 (1.41)	54.9%	11.5%	20 (14.4%)	8 (7.6%)
I felt that the treatment was not motivating	10.7%	2.41 (1.26)	69.2%	7.4%	11 (7.9%)	7 (6.7%)

Bold numbers indicate significant differences between patients with terminated treatment and those still in psychotherapy.

Some negative effects were generally uncommon but commonly related to treatment, including dependency on the therapist, feeling ashamed because of the treatment, or demoralisation. Slightly fewer than one-fifth reported problems in understanding the treatment or the therapist. Of the full sample, 56.6% reported having had experienced *any negative effect caused by their psychological treatment* (83.2% reported any effect, not only those related to the therapy).

Table 3 also contrasts negative effect rates for patients with completed treatment and those still in psychotherapy. Those who terminated treatment reported two items at a higher rate (‘I felt more worried’ and ‘Unpleasant memories resurfaced’; χ² > 4.06, *P* < 0.05; 0.165 > Cramer's V > 0.129).

To test the relationship between negative effects (number of negative effects related to therapy) and the subscales of the HAQ, correlations were calculated. Significant but low/moderate correlation relationships were observed between negative effects and therapeutic progress (*r* = −0.160, *P* < 0.05) and between negative effects and quality of the relationship (*r* = −0.383, *P* < 0.01).

As the NEQ does not cover indicators of malpractice and boundary violations, we used the six relevant items from the INEP. With one exception (feeling violated by statements of the therapist, 16.8%), agreement to these items was rather low. Sixteen (6.5%) *v*. 14 (5.7%) individuals indicated that they felt the therapist forced them to do things that they did not want to do (e.g. exposure) or felt the therapist was making fun of them. Sexual assault, physical assault and violation of confidentiality were reported by one individual each.

## Discussion

Based upon the conclusion that our knowledge about negative effects of psychotherapy is still limited,^1–6^ one of the unmet needs is sufficient study of the type and quantity of negative effects of psychotherapy under naturalistic conditions. There are several approaches to reach the goal of acquiring more detailed data concerning negative effects. For example, Crawford^5^ approached psychotherapeutic services in England and Wales to survey patients receiving treatment within these services. Although this approach might result in a population close to being representative of psychotherapy patients in a specific health system, it would not be representative of the wider population.

Another approach would be to start by drawing a random sample from a national population and to filter those individuals who had received psychotherapeutic treatment in a certain time period. The latter approach was chosen in a study of Albani et al^18–20^ related to the German population. In contrast to our survey, that study focused on formal characteristics of psychotherapies, patients’ experiences with choosing and finding a therapist, and general figures related to the effectiveness of psychotherapy from the patients’ perspective. In their survey, Albani et al asked only a very small number of questions related to general opinions about the patients’ psychotherapists and did not explicitly focus on negative effects. The sampling method of the Albani et al study probably did not yield a sample representative of psychotherapy patients in Germany. On the other hand, by avoiding direct selection of these patients, the procedure likely resulted in an unbiased sample from which patient experiences could be derived.

So far, data related to the prevalence of psychotherapeutic change, change rates and the occurrence of negative effects are quite variable and do not allow aggregation owing to the different data sources and measures. To add data from a representative population, our study followed the model of Albani et al, selecting individuals from a random national sample of the German population and determining which had been treated with psychotherapy. This resulted in a sample of 244 individuals who were interviewed in detail – in contrast to Albani et al – with a focus on effectiveness, helping alliance and a description of negative effects.

In fact, the resulting sample had quite similar characteristics to those of the German population. The ratio of males to females appeared to be more balanced in our sample than in the Albani study and closer to the distribution of the national population. In a large clinical sample of German out-patients,^30^ the percentage of female patients was much higher than in Albani's study^19,20^ (77%), showing that the general population is different from the population using the psychotherapeutic system. Individuals in the under-45 age group were underrepresented whereas those of 45 to 65 years of age were overrepresented in our sample, compared with the general population. Compared with the national population, individuals in our sample had a higher educational level. This probably reflects selective mechanisms of patients’ access to the psychotherapeutic system.^31^

Of the initial sample, 7.44% indicated experiences with psychotherapy during the prior 6 years. Although there are no exact estimates of the proportion of individuals seeking psychotherapeutic treatment in Germany, there are some figures for this percentage that can be used to for comparison. Rommel et al^32^ reported that 11.3% of German females and 8.1% of males over 18 years of age sought psychotherapeutic *or* psychiatric help over the course of 1 year (Survey Health in Germany). A study of adult health in Germany^33^ reported that 5.3% of females and 3.2% of males between 18 and 79 years of age made use of psychotherapy in the public health system (i.e. attending licensed therapists with reimbursement of the costs by health insurance). Based on these comparative figures, we think that our sample reflects a realistic proportion of psychotherapy users.

Based on the data obtained in our interview study with the final sample of 244 (former) psychotherapy patients, we found a relatively positive evaluation of the therapeutic relationship using the HAQ,^22^ which was comparable to that found by other studies. The reports of our sample were generally positive regarding the quality of the working alliance and trust in the therapeutic relationship. At least 80% of all individuals agreed at least to some extent with the positive formulations of the HAQ.

On the other hand, there were some indicators of problems in the therapeutic relationship. One of the most prominent indicators was the report that at least 19% thought that the treatment would not help and ended their therapy prematurely. Also of relevance is the finding that in 24.2% of those cases, the end of treatment was a proposal of the therapist. Although we have no information on whether these were negotiated or unilateral decisions, this finding raises concerns about the lack of participatory decision-making about when to end therapy.

Although we did not use standardised scales that are commonly used to assess treatment outcomes, our data suggest that ‘direct measurements’ of different fields susceptible to psychotherapeutic change indicate improvement rates between 26.6% and 67.6%. The improvement rates of common outcomes (i.e. interactions with others, improvement in depressed mood, personal development), reported by more than 60% of the individuals, particularly demonstrate that the sample might be representative of psychotherapy patients, as similar rates are reported in the research literature.^12^

The improvement rates in our sample are also similar to those reported by Albani et al, with respect to both change rates and rates of deterioration as well as differences between single areas of change. However, the improvement rates in the Albani study (with a larger sample) were somewhat higher than those in our sample. For example, an improvement in depressed mood was reported by 67.6% of our sample and by 78.6% in the Albani study. The general evaluations of the treatments were also in line with those reported by Albani et al.

The primary focus of our study was an estimation of negative effects (or side-effects as negative effects paralleling correct treatment in the sense of Linden's classification^3^) of psychotherapies, with the NEQ as the core instrument. Twenty different negative effects could be attributed to the treatment or to other causes.

The survey results reported in our sample are comparable with those reported in different clinical samples with the NEQ; we found similar results to those of other studies using this method in different samples and psychotherapeutic settings. In a recent study, Rozental et al^2^ reported: ‘As for the rate of negative effects, the number of participants reporting negative effects in the current study was 50.9%, consistent with 58.7% among patients in a psychiatric setting who responded to the INEP’.^34^ However, this number varies significantly between investigations, with rates as high as 92.9% among patients with obsessive–compulsive disorder assessed with the Side-effects of Psychotherapy Scale in a study by Moritz et al,^35^ and as low as 5.2% in a national survey by Crawford et al^5^ probing for ‘lasting bad effects from the treatment’. Hence, different studies assess a range of negative effects, from transient ‘side-effects’ to lasting harm, making it difficult to compare ratios directly. Even within a subtype of negative effect, different methods of assessment will yield different results, so accurate estimates are not yet available.

Finally, since we had limited resources, we restricted our investigation of malpractice and boundary violations in psychotherapy in this study to only the six items of the INEP. These items form a subscale of the instrument mainly developed to cover side-effects of psychotherapeutic interventions. In general, in our sample, the rates of boundary violations were very low, even lower than one would have estimated from the specific studies in this field. For example, Becker-Fischer and Fischer^36^ reported rates of sexual boundary violations in psychotherapies that were much higher than 5%, whereas in our sample such violations occurred in three of the 244 cases (1.2%).

### Strengths and limitations

The main strength of this study was clearly the sampling procedure, which started with a large (>5000) sample representative of the German population and then sought to find individuals disclosing experience with psychotherapy in the German health system, currently or during the past 6 years. We used some of the items from a former survey focusing on more general aspects of psychotherapy and added (parts of) instruments specifically developed to capture negative effects (NEQ) or malpractice (INEP). These additions have shown good psychometric qualities in this and other studies and allow comparisons with other studies or sampling procedures. Compared with other studies, e.g. the Crawford et al^5^ survey, we obtained much more detailed results on negative effects as opposed to global ‘lasting bad effects’.

Despite our best efforts, the final sample of 244 was rather small, although it was within the expected range for the use of psychotherapy in the population. Another limitation was the fact that 98 of the 244 participants were surveyed, on average, 2.63 years after completion of their psychotherapy. Of the 244, 139 had already completed their psychotherapy, among whom 98 provided the date of the end of therapy. Thus, the results may have been biased by recall effects. More specifically, there may have been a tendency to only remember adverse aspects of the treatment and neglect the positive ones, or to forget certain unwanted events that occurred several years ago. However, comparisons between those currently undergoing psychological treatment and those remembering their treatment retrospectively yielded only minor differences with respect to both general evaluations of psychotherapy and negative effects.

Moreover, as only 65% of eligible participants accepted the invitation to the interview, the results could be open to selection bias. For example, participants who were unhappy about their treatment might be more (or less) likely to respond to a study on the effects of psychotherapy or might exaggerate negative effects experienced during psychotherapy.

A comparison of demographic data from the recruited sample and the final sample revealed some minor differences regarding age distribution and educational level. However, participants were not recruited only on the basis of potential experiences of negative effects, as positive aspects of treatments were evaluated as well, limiting the risk of selection bias. Also, the response rate in our study was similar to those of other studies on negative effects of psychotherapy, which found rates of 59%^6^ and 61%^29^; it was even much higher than the rate of 19% found in one study.^5^

Our results related to problematic issues such as boundary violations should encourage a detailed examination of patient complaints. So far, these have been mainly reported by certain institutions who serve as receiving agencies for psychotherapy-related complaints.^37,38^

In the future, more research on the prevalence of negative effects would be useful. This would include a more systematic assessment of these effects in clinical trials.^8^ It would be interesting to try to recruit a similar sample as that used in our study to estimate the occurrence of more subtle violations of borders and other problematic issues in psychotherapy. According to the studies relating to such complaints, these are much more common than severe ethical problems such as a sexual assault in the treatment room. Addressing such violations and intensifying the more general focus on negative effects would eventually enrich training, supervision and clinical practice with the goal of avoiding harm in psychotherapy.

## Data Availability

The data that support the findings of this study are available from the corresponding author, B.S., upon reasonable request.

## Supplementary material

For supplementary material accompanying this paper visit http://dx.doi.org/10.1192/bjo.2021.1025. click here to view supplementary material

## References

[1] Parry GD, Crawford MJ, Duggan C. Iatrogenic harm from psychological therapies – time to move on. Br J Psychiatry 2016; 208: 210–2. 26932481 10.1192/bjp.bp.115.163618

[2] Rozental A, Kottorp A, Forsström D, Mánsson K, Boettcher J, Anderson G, The negative effects questionnaire: psychometric properties of an instrument for assessing negative effects in psychological treatments. Behav Cogn Psychother 2019; 47: 559–72. 30871650 10.1017/S1352465819000018

[3] Linden M. How to define, find and classify side effects in psychotherapy: from unwanted events to adverse treatment reactions. Clin Psychol Psychother 2013; 20: 286–96. 22253218 10.1002/cpp.1765

[4] Herzog P, Lauff S, Rief W, Brakemeier E-L. Assessing the unwanted: a systematic review of instruments used to assess negative effects of psychotherapy. Brain Behav 2019; 9: e01447. 31647202 10.1002/brb3.1447 PMC6908878

[5] Crawford MJ, Thana L, Farquharson L, Palmer L, Hancock E, Bassett P, Patient experience of negative effects of psychological treatment: results of a national survey. Br J Psychiatry 2016; 208: 260–5. 26932486 10.1192/bjp.bp.114.162628

[6] Gerke L, Meyrose A-K, Ladwig I, Rief W, Nestoriuc Y. Frequencies and predictors of negative effects in routine inpatient and outpatient psychotherapy: two observational studies. Front Psychol 2020; 11: 2144. 32982878 10.3389/fpsyg.2020.02144 PMC7478145

[7] Linden M, Strauß B, Scholten S, Nestoriuc Y, Brakemeier E-L, Wasilewski J. Definition und Entscheidungsschritte in der Bestimmung und Erfassung von Nebenwirkungen von Psychotherapie [Definition and decision-making in the determination and detection of side effects of psychotherapy]. Psychother Psych Med 2018; 68: 377–82. 10.1055/a-0619-5949 30286505

[8] Klatte R, Strauss B, Flückiger C, Rosendahl J. Adverse effects of psychotherapy: protocol for a systematic review and meta-analysis. System Rev 2018; 7: 135. 30193585 10.1186/s13643-018-0802-x PMC6128985

[9] Scott J, Young AH. Psychotherapies should be assessed for both benefit and harm. Br J Psychiatry 2016; 208: 208–9. 26932480 10.1192/bjp.bp.115.169060

[10] Strupp HH. The Vanderbilt psychotherapy studies: synopsis. J Consult Clin Psychol 1993; 61(3): 431–3. 8326043 10.1037//0022-006x.61.3.431

[11] Linden M, Strauss B. Risiken und Nebenwirkungen von Psychotherapie [Risks and side effects of psychotherapy] (2nd edn). WMV, 2018. 10.1055/a-0630-3297 30286504

[12] Lambert MJ. Maximizing psychotherapy outcome beyond evidence-based medicine. Psychother Psychosom 2017; 86(2): 80–89. 28183083 10.1159/000455170

[13] Barlow DH. Negative effects from psychological treatments. a perspective. Am Psychol 2010; 65: 13–20. 20063906 10.1037/a0015643

[14] Mohr DC. Negative outcome in psychotherapy: a critical review. Clin Psychol Sci Prac 1995; 2: 1–27.

[15] Dimidjian S, Hollon SD. How would we know if psychotherapy were harmful? Am Psychol 2010; 65: 21–33. 20063907 10.1037/a0017299

[16] Lilienfeld SO. Psychological treatments that cause harm. Perspect Psychol Sci 2007; 2: 53–70. 26151919 10.1111/j.1745-6916.2007.00029.x

[17] Castonguay LG, Boswell JF, Constantino MJ, Goldfried MR, Hill CE. Training implications of harmful effects of psychological treatments. Am Psychol 2010; 65: 34–49. 20063908 10.1037/a0017330

[18] Albani C, Blaser G, Geyer M, Schmutzer G, Goldschmidt S, Brähler E. Wer nimmt in Deutschland ambulante Psychotherapie in Anspruch? [Who is using outpatient psychotherapy in Germany]. Psychother Psych Med 2009; 59: 281–3. 10.1055/s-0028-1103267 19274608

[19] Albani C, Blaser G, Geyer M, Schmutzer G, Brähler E. Ambulante Psychotherapie in Deutschland aus Sicht der Patienten [Outpatient psychotherapy in Germany from the patients’ point of view – Part 1]. Psychotherapeut 2010; 55: 503–14.

[20] Albani C, Blaser G, Geyer M, Schmutzer G, Brähler E. Ambulante Psychotherapie in Deutschland aus Sicht der Patienten [Outpatient psychotherapy in Germany from the patients’ point of view – part 2]. Psychotherapeut 2011; 56: 51–60.

[21] Kish L. A procedure for objective respondent selection within the household. J Am Stat Assoc 1949; 44: 380–7.

[22] Bassler M, Potratz B, Krauthauser H. Der “Helping Alliance Questionnaire” (HAQ) von Luborsky. Möglichkeiten zur Evaluation des therapeutischen Prozesses von stationärer Psychotherapie [The “Helping Alliance Questionnaire” (HAQ) by Luborsky]. Psychotherapeut 1995; 40: 23–32.

[23] Nübling C, Kraft M, Henn J, Kriz D, Lutz W, Schmidt J, Bassler M, Testing the psychometric properties of the Helping Alliance Questionnaire (HAQ) in different health care settings. Psychother Psych Med 2017; 67: 465–76. 10.1055/s-0043-111083 28854445

[24] Ladwig I, Rief W, Nestoriuc Y. Welche Risiken und Nebenwirkungen hat Psychotherapie? – Entwicklung des Inventars zur Erfassung negativer Effekte von Psychotherapie (INEP) [What are the risks and side effects to psychotherapy? Development of an inventory for the assessment of negative effects of psychotherapy (INEP) ]. Verhaltenstherapie 2014; 24: 252–63.

[25] Federal Statistical Office of Germany. Bevölkerung in Deutschland I [German Population I]. Federal Statistical Office of Germany, 2019 (https://de.statista.com/statistik/studie/id/7661/dokument/bevoelkerung-in-deutschland-i-statista-dossier/ [cited 5 Feb 2021]).

[26] Federal Statistical Office of Germany. Bevölkerung nach Bildungsabschluss [Population in Germany According to Educational Level]. Federal Statistical Office of Germany, 2019 (https://www.destatis.de/DE/Themen/Gesellschaft-Umwelt/Bildung-Forschung-Kultur/Bildungsstand/_inhalt.html#sprg234412 [cited 5 Feb 2021]).

[27] Federal Statistical Office of Germany. Bevölkerung und Erwerbstätigkeit [Population in Germany According to Occupation], Fachserie 1, Reihe 4.1. Federal Statistical Office of Germany, 2019 (https://www.destatis.de/DE/Service/Bibliothek/_publikationen-fachserienliste-1.html [cited 5 Feb 2021]).

[28] Federal Statistical Office of Germany. Bevölkerung nach Familienstand [Population in Germany According to Family Status]. Federal Statistical Office of Germany, 2019 (https://www.destatis.de/DE/Themen/Gesellschaft-Umwelt/Bevoelkerung/Bevoelkerungsstand/Tabellen/familienstand-jahre-5.html [cited 5 Feb 2021]).

[29] Federal Statistical Office of Germany. Einkommen Privater Haushalte [Income of Private Households]. Federal Statistical Office of Germany, 2019 (https://www-genesis.destatis.de/genesis/online?operation=table&code=63121-0003&bypass=true&levelindex=0&levelid=1612802491776 [cited 5 Feb 2021]).

[30] Altmann U, Zimmermann A, Kirchmann HA, Kramer D, Fembacher A, Bruckmayer E, Outpatient psychotherapy reduces health-care costs: a study of 22,294 insurants over 5 years. Front Psychiatry 2016; 7: 98. 27378950 10.3389/fpsyt.2016.00098 PMC4904013

[31] Strauß B. Chancenungleichheit auf der Suche nach einem Therapieplatz: Schlussfolgerungen für die zukünftigen Aufgaben der Psychotherapieforschung [Inequivalent opportunities to find psychotherapeutic treatment: conclusions for future tasks of psychotherapy research]. Psychotherapeut 2015; 60(5): 389–96.

[32] Rommel A, Bretschneider J, Kroll LE, Prütz F, Thom J. Inanspruchnahme psychiatrischer und psychotherapeutischer Leistungen [Use of psychiatric and psychotherapeutic care]. J Health Monit 2017; 2(4): 3–23.

[33] Rattay P, Butschalowsky HG, Rommel A, Prütz F, Jordan S, Nowossadeck E, Utilization of outpatient and inpatient health services in Germany. results of the German health interveiw and examination survey for adults (DEGS1). Bundesgesundheitsbl 2013; 56: 832–44. 10.1007/s00103-013-1665-x 23703505

[34] Rheker J, Beisel S, Kräling S, Rief W. Rate and predictors of negative effects of psychotherapy in psychiatric and psychosomatic inpatients. Psychiatry Res 2017; 254: 143–50. 28460285 10.1016/j.psychres.2017.04.042

[35] Moritz S, Fieker M, Hottenrott B, Seeralan T, Cludius B, Kolbeck K, No pain, no gain? Adverse effects of psychotherapy in obsessive-compulsive disorder and its relationship to treatment gains. J Obsess Compuls Relat Disord 2015; 5: 61–6.

[36] Becker-Fischer M, Fischer G. Sexuelle Übergriffe in der Psychotherapie [Sexual assaults in psychotherapy]. Asanger, 2008.

[37] Khele S, Symons C, Wheeler S. An analysis of complaints to the British association for counselling and psychotherapy, 1996–2006. Couns Psychother Res 2008; 8: 124–32.

[38] Kaczmarek S, Passmann K, Cappel R, Hillebrand V, Schleu A, Strauss B. Wenn Psychotherapie schadet. Eine qualitative und quantitative Untersuchung von Beschwerden über psychotherapeutische Behandlungen [When psychotherapy hurts. A qualitative and quantitative study of complaints about psychotherapeutic treatments]. Psychotherapeut 2012; 57: 402–9.

