# Supplementary material for: Negative effects of psychotherapy: estimating the prevalence in a random national sample
Source: BJPsych Open. 2021 Oct 4;7(6):e186. doi: 10.1192/bjo.2021.1025 (PMC8503914; doi:10.1192/bjo.2021.1025)
Supplement: Supplementary file 1 [file S2056472421010255sup001.docx]

Table S1. Summary of the individuals´ answers to the 11 HAQ–questions (n/%; Mean and SD)

|  | Agree | Disagree |  |
| --- | --- | --- | --- |
| 1 I believe(d) that my therapist helps me | n = 218  (89,3%) | n = 26  (10,7%) | M = 1,93  SD = 1,21 |
| 2 I feel/felt that the treatment helps me | n = 217  (88,9%) | n = 25  (10,2%) | M = 1,95  SD = 1,25 |
| 3 I have gained new perspectives | n = 213  (87,3%) | n = 28  (11,5%) | M = 2,12  SD = 1,32 |
| 4 Due to the therapy I am feeling better | n = 220  (90,2%) | n = 24  (9,8%) | M = 1,95  SD = 1,20 |
| 5 I can/could see that I will solve the problems that lead me into treatment | n = 197  (80,7%) | n = 46  (18,9%) | M = 2,49  SD = 1,45 |
| 6 I have/had the feeling that I can rely upon my therapist | n = 219  (89,8%) | n = 23  (9,4%) | M = 1,69  SD = 1,21 |
| 7 I have/had the feeling that my therapist understands me | n = 221  (90,6%) | n = 22  (9,0%) | M = 1,72  SD = 1,15 |
| 8 I have/had the feeling that I will be able to reach my goals | n = 226  (92,6%) | n = 17  (7,0%) | M = 1,60  SD = 1,05 |
| 9 I have/had the feeling that my therapist and I pull together | n = 218  (89,3%) | n = 25  (10,2%) | M = 1,82  SD = 1,22 |
| 10 I believe my therapist and I have similar ideas about the nature of my problems | n = 207  (84,8%) | n = 31  (12,7%) | M = 2,08  SD = 1,21 |
| 11 I trust that I will understand myself and continue to deal with myself even if I will have no more sessions with my therapist | n = 221  (90,6%) | n = 20  (8,2%) | M = 1,98  SD = 1,08 |

*Note*. In order to report frequencies for the HAQ more stringently, items were converted into a dichotomous variable according to how strongly people agree or disagree regarding different statements about their relationship to the therapist. Items 1, 6, 7, 8, 9 and 10 comprise the quality of therapeutic relationship subscale and items 2, 3, 4, 5 and 11 represent the satisfaction with therapeutic outcome subscale.
